# The Role of NLRP3 Inflammasome in Diabetic Cardiomyopathy and Its Therapeutic Implications

**DOI:** 10.1155/2022/3790721

**Published:** 2022-09-06

**Authors:** Kai Ding, Chao Song, Hengjing Hu, Kai Yin, Hong Huang, Huifang Tang

**Affiliations:** ^1^Department of Cardiology, The First Affiliated Hospital, Hengyang Medical School, University of South China, Hengyang, Hunan Province 421001, China; ^2^Cardiovascular Lab of Big Data and Imaging Artificial Intelligence, Hengyang Medical School, University of South China, Hengyang, Hunan Province 421001, China; ^3^Institute of Cardiovascular Disease, The First Affiliated Hospital, Hengyang Medical School, University of South China, Hengyang, Hunan Province 421001, China; ^4^Clinical Research Center for Myocardial Injury in Hunan Province, Hengyang, Hunan Province 421001, China; ^5^Guangxi Key Laboratory of Diabetic Systems Medicine, Guilin Medical University, Guilin, Guangxi 541000, China

## Abstract

Diabetic cardiomyopathy (DCM) is a serious complication of diabetes mellitus (DM). However, the precise molecular mechanisms remain largely unclear, and it is still a challenging disease to diagnose and treat. The nucleotide-binding oligomerization domain and leucine-rich repeat pyrin 3 domain (NLRP3) inflammasome is a critical part of the innate immune system in the host to defend against endogenous danger and pathogenic microbial infections. Dysregulated NLRP3 inflammasome activation results in the overproduction of cytokines, primarily IL-1*β* and IL-18, and eventually, inflammatory cell death-pyroptosis. A series of studies have indicated that NLRP3 inflammasome activation participates in the development of DCM, and that corresponding interventions could mitigate disease progression. Accordingly, this narrative review is aimed at briefly summarizing the cell-specific role of the NLRP3 inflammasome in DCM and provides novel insights into developing DCM therapeutic strategies targeting the NLRP3 inflammasome.

## 1. Introduction

Diabetic cardiomyopathy (DCM) is a specific cardiac phenotype in diabetic patients that is characterized by cardiac structural changes (cardiac hypertrophy, cardiomyocyte death and fibrosis) and functional abnormalities in the absence of hypertension, coronary atherosclerosis, and significant valvular heart diseases. Epidemiological data demonstrate that the prevalence of DCM is increasing in parallel with the incredibly fast worldwide spread of diabetes mellitus (DM). The incidence of DCM is estimated to be approximately 1.1% in community-based populations and 16.9% in diabetic patients [1], and more importantly, DCM is one of the leading causes of mortality among the various complications of DM. Numerous studies on the pathophysiology of DCM have demonstrated the importance of insulin resistance, cardiac metabolic disorders, oxidative stress, abnormal coronary microcirculation, cardiac autonomic neuropathy, and maladaptive inflammatory responses [2, 3]. However, few diagnostic tools are currently available, and no formal guidelines or approved specific pharmacotherapeutics for DCM have been established, suggesting that understanding the molecular mechanisms leads to underdiagnosis and limits the prevention and treatment of this condition. Therefore, further exploring the underlying mechanisms of DCM is helpful to develop early prevention strategies to reduce the morbidity and mortality of DCM.

Currently, there is consensus that systemic and local maladaptive inflammation-mediated mechanisms contribute considerably to pathogenesis and progression in this regard [4, 5]. In the heart, chronic inflammation in cardiomyocytes, cardiac fibroblasts (CFs), and coronary artery endothelial cells (CAECs) leads to increased ventricular stiffness and asymptomatic diastolic dysfunction, followed by cardiac remodeling, myocardial hypertrophy, and impaired cardiac contractile function, eventually resulting in clinical heart failure (HF) [6]. The NLRP3 inflammasome is a critical part of the innate immune system that initiates and propagates inflammatory responses in host defense against endogenous danger and pathogenic microbial infections immediately [7]. The NLRP3 inflammasome is a double-edged sword in various diseases, especially in sterile inflammatory diseases, and the outcomes can be either good or bad depending on the disease and the genetic background [8]. Emerging evidence highlights the involvement of NLRP3 inflammasome-mediated inflammation in the initiation and progression of metabolic disorders [9, 10] and cardiovascular diseases [11–13]. Consistent results have been shown in various animal models [14, 15] which indicate that the NLRP3 inflammasome links cardiometabolic to immune function and inflammation. Furthermore, the NLRP3 inflammasome is highly expressed not only in immune cells but also in cardiomyocytes, CFs, and CAECs [16]. The specific pathology of the NLRP3 inflammasome in the heart suggests that it has emerged as a strong candidate to bridge inflammation and DCM, which is activated by ROS, hyperglycemia, hyperlipidemia, and advanced glycation end products during DCM. It triggers inflammation and promotes subcellular alterations in different cardiac cells, leading to cardiac injury, cell death, and maladaptive myocardial remodeling, and this effect is independent of the species and strains of experimental animals [17–19]. The evidence together reveals a deleterious role of the NLRP3 inflammasome in the development and progression of DCM, providing novel insight into developing therapeutics for DCM.

The present review summarizes the effect of the NLRP3 inflammasome on the major cellular components of the heart, such as cardiomyocytes, CFs, and CAECs, to clarify its role in DCM. We also focus on the underlying mechanism of the NLRP3 inflammasome in DCM and discuss several promising therapeutic strategies targeting the NLRP3 inflammasome to provide a new perspective for the prevention and treatment of DCM in the future.

## 2. NLRP3 Inflammasome Overview

### 2.1. Molecular Compositions and Structure of the NLRP3 Inflammasome

The NLRP3 inflammasome consists of three parts, namely, the sensor molecule NLRP3 protein, the adapter apoptosis-associated speck-like protein containing a caspase recruitment domain (ASC), and the effector procysteinyl aspartate specific proteinase-1 (procaspase-1) [20]. Sensor protein includes a carboxyl (C) terminal, a central, and an amino acid (N) terminal domain. The C-terminus and the central domain are responsible for the recognition of corresponding ligands and oligomerization, respectively. The N-terminal part contains a pyrin domain (PYD) that is capable of recruiting ASC to mediate downstream signaling transmission. ASC is a bilateral adaptor protein containing both a PYD and a caspase-recruitment domain (CARD); therefore, it is able to bridge NLRP3 protein and procaspase-1 [21]. Upon activation of the NLRP3 inflammasome sensor molecule, activated NLRP3 protein recruits the multiple adaptor ASC filaments coalescing into an ASC speck via homotypic PYD–PYD interaction. Therefore, ASC serves as a recruitment point for procaspase-1 through CARD. Subsequently, these three proteins form a multiprotein complex, which leads to caspase dimerization and directly promotes proteolytic cleavage of inactive procaspase-1 into active caspase-1 [22]. Consequently, caspase-1, as an effector protein, processes interleukin precursors pro-IL-1*β*/18 to mature forms proinflammatory IL-1*β*/18 and cleaves gasdermin D (GSDMD) to generate an N-terminal cleavage product (GSDMD-NT). GSDMD-NT provokes a proinflammatory and lytic mode of cell death termed pyroptosis by distributing across the cell membrane and forming cytotoxic pores, thereby releasing inflammatory mediators and damaged DNA [23]. In conclusion, the homologous interactions of domains are the structural basis for NLRP3 inflammasome assembly. The NLRP3 inflammasome can function as an inflammatory player after its assembly. The NLRP3 inflammasome conditions and biological outcomes in animal and cell models of DCM were summarized in Table 1.

### 2.2. Molecules and Mechanisms Involved in Activation of the NLRP3 Inflammasome

**Figure 1:**
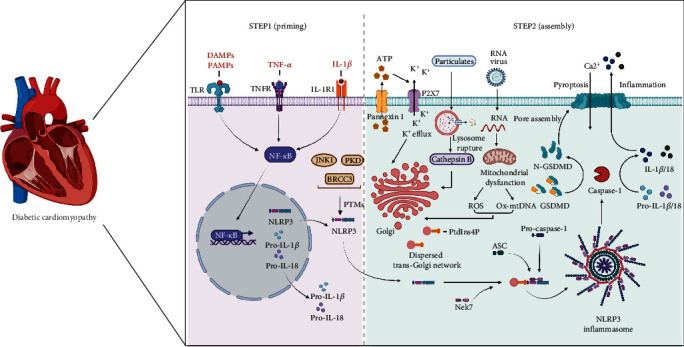
NLRP3 inflammasome two-step mechanism. NLRP3 inflammasome activation requires a two-step mechanism. In step 1, namely, priming, NF-*κ*B signaling is activated by specific lines recognized by TLR, TNFR, and IL-1R1, leading to increased transcription of the NLRP3 components pro-IL-1b and pro-IL-18. In step 2, assembly, ATP, pathogen-associated RNA, particulates, and other stresses induce molecular and cellular signaling events, including ion flux, mitochondrial dysfunction, reactive oxygen species (ROS) production, lysosomal membrane rupture and subsequent cathepsin B leakage, and oxidized mitochondrial DNA release. In turn, cellular homeostasis imbalance contributes to disassembly of the trans-Golgi network (TGN) and NLRP3 activation, inducing Nek7 and PTMs of NLRP3. The dispersed trans-Golgi network (dTGN) serves as a scaffold for active NLRP3 via PtdIns4P, thereby leading to recruitment of the adaptor protein ASC and the effector protein procaspase-1. These three proteins form a multiprotein complex, which leads to caspase-1 activation, which in turn processes interleukin precursors pro-IL-1*β*/18 to mature forms proinflammatory IL-1*β*/18 and cleaves gasdermin D (GSDMD) to generate an N-terminal cleavage product (GSDMD-NT). GSDMD-NT provokes pyroptosis, and these cytokines are then released into the extracellular space—ASC: apoptosis-associated speck-like protein, ATP: adenosine triphosphate, BRCC3: BRCA1/BRCA2-containing complex subunit 3, IL-1R1: interleukin-1 receptor type 1, JNK1: Jun N-terminal kinase-1, Nek7: NIMA-related kinase 7, Ox-mtDNA: oxidized mitochondrial DNA, PKD: protein kinase D, PTMs: posttranslational modifications, PtdIns4P: phosphatidylinositol-4-phosphate, ROS: reactive oxygen species, TLR: Toll-like receptor, and TNFR: TNF receptor.

NLRP3 inflammasome activation necessitates two sequential procedures, namely, priming and assembly. In the priming phase, pattern recognition receptors, TNF receptors, and IL-1 receptors recognize the corresponding priming signals (including pathogen-associated molecular patterns, damage-associated molecular patterns, TNF-*α*, and IL-1*β*), thereby promoting NF-*κ*B transcription and enhancing the production of NLRP3, pro-IL-1*β*, and pro-IL-18 [35, 36]. Sequentially, NLRP3 undergoes posttranslational modifications (PTMs) and binds to NIMA-related kinase 7 (Nek7) to form the NLRP3-Nek7 complex, which becomes activated. PTMs and the formation of the NLRP3-Nek7 complex license the NLRP3 protein to rapidly assemble with other components to form a complex in response to stimulatory signals [21, 37] (Figure 1). The mechanisms for the PTMs of NLRP3 have been investigated in depth. NLRP3 is readily ubiquitinated and subsequently degraded in proteasomes at steady state unless this is actively suppressed by NLRP3 deubiquitination in the presence of NLRP3-inducing stimuli [38]. Furthermore, SUMOylation, phosphorylation, and acetylation of the NLRP3 protein also play an important role in regulating the activity of the NLRP3 inflammasome [37, 39, 40]. The potential mechanisms may be related to PTMs altering the net charge of NLRP3 or spatially inhibiting interactions with scaffolds [41]. These findings reveal the importance of PTMs of NLRP3 in mediating NLRP3 inflammasome activity and its potential application in the treatment of NLRP3 inflammasome-related diseases.

In the assembly phase, the activated NLRP3 protein can recognize a broad range of seemingly unrelated stimuli, including particles, endotoxin, pathogen-associated RNA, and ATP, which triggers the assembly of NLRP3, ASC, and procaspase-1 into an activated macromolecular multiprotein complex. Therefore, the complex promotes the production of inflammatory cytokines and determines cell fate for pathogen or cellular stress surveillance. However, due to the structural diversity of these stimulators, direct interaction between stimuli and NLRP3 seems unlikely [20]. Indeed, NLRP3 agonists have been shown to activate NLRP3 inflammasomes by triggering multiple molecular and cellular signaling events, including ion flux, mitochondrial dysfunction, reactive oxygen species (ROS) production, lysosomal membrane rupture and subsequent cathepsin B leakage, and oxidized mitochondrial DNA [42]. However, a common stress signal caused by these diverse events remains unknown. A recent landmark study by Chen revealed that different NLRP3 activators lead to disassembly of the trans-Golgi network (TGN). NLRP3 is recruited to the dispersed TGN (dTGN) through ionic bonding, and then the dTGN serves as a scaffold for NLRP3 aggregation into multiple puncta, leading to polymerization of the adaptor protein ASC, thereby activating downstream signaling cascades [43]. This work demonstrates that dTGN is the common stress event that is required for NLRP3 activation in response to diverse agonists. This has incrementally improved our understanding of the molecular mechanism of NLRP3 activation. However, how NLRP3 activators initiate the dispersion of the TGN is unclear and needs further clarification.

## 3. Cell-Specific Roles of the NLRP3 Inflammasome in DCM

### 3.1. NLRP3 Inflammasome in Cardiomyocytes

Cardiomyocyte death, either progressive or acute, has been considered to be a fundamental pathological process in multiple cardiovascular diseases ranging from heart failure to myocardial infarction to DCM. Pyroptosis is a novel form of programmed cell death that consists of canonical pathways and noncanonical pathways and has been regarded as a proinflammatory and uncontrolled type of cell death. NLRP3 inflammasome-mediated caspase-1-dependent pyroptosis is a canonical pathway. Emerging evidence has verified that NLRP3 inflammasome-mediated cardiomyocyte pyroptosis is a key participant in DCM [24]. Human diabetic hearts show upregulation of NLRP3 inflammasome activation and cardiac pyroptosis when compared with nondiabetic heart tissues, and in human ventricular cardiomyocytes, hyperglycemia (35 mM glucose) apparently induces increased NLRP3, caspase-1, and IL-1*β* protein expression, concomitantly accompanied by obvious cardiomyocyte pyroptosis [25], thereby leading to a loss of contractile units and cardiac dysfunction. These findings confirm the distinctive role of NLRP3 inflammasome-mediated pyroptosis in DCM. In diabetic rats, the expression of NLRP3 and IL-1*β* increases significantly in heart tissue compared with nondiabetic controls, while silencing the NLRP3 gene ameliorates cardiac inflammation and pyroptosis and improves cardiac function in both diabetic rat model and H9c2 cardiomyocytes incubated with high glucose [26]. This study highlights that targeting the NLRP3 inflammasome would play a therapeutic role in cell death during DCM. Moreover, observations indicate that regulation of NLRP3 inflammasome activity and its mediated cardiac pyroptosis through the Sirt3 signaling pathway modulates the occurrence and development of DCM [44, 45]. An increasing amount of evidence demonstrates that noncoding RNAs (ncRNAs) are important epigenetic regulators of the immune response in the heart and are critically involved in the molecular mechanisms of DCM [46]. NcRNAs influence the susceptibility to the pathogenesis of diabetic cardiomyopathy by mediating NLRP3 inflammasome activation-induced pyroptosis [27, 28, 30]. Moreover, although multiple aspects of epigenetic regulation, from DNA modification to protein modification, have been extensively studied in DCM, the role of RNA modification in DCM is just beginning to be elucidated. Total N6-methyladenosine (m6A) methylation modification levels are higher in DCM [47]. Recent studies have shown that the mRNA methyltransferase METTL14 specifically induces an increase in m6A methylation of NLRP3, thereby increasing the expression level of NLRP3 protein, while downregulation of m6A methylation of NLRP3 mRNA by targeting METTL14 can prevent pyroptosis in nucleus pulposus cells [48]. These findings provide valuable insights into the pathomechanism of DCM and imply that modification of epitranscriptomic processes, such as m6A, is a potentially interesting therapeutic approach.

In addition to cell loss, cardiomyocyte hypertrophy is an important characteristic of cardiomyocytes in DCM. Myocardial hypertrophy is a pathological stage of various cardiovascular diseases, and it is an adaptive and compensatory mechanism to maintain contractile function in response to various stimulations. However, maladaptive myocardial hypertrophy induced by chronic long-term stimulation results in myocardial structural disorders and cardiac insufficiency. Numerous cytokines produced by cardiomyocytes, fibroblasts, and immune cells are related to myocardial hypertrophy and heart failure [49]. Circulating IL-1 levels are elevated in patients with pathological hypertrophy and heart failure [50, 51]. Clinical studies of IL-1 signal blockade (the IL-1 receptor antagonist anakinra and the IL-1*β* neutralizing antibody canakinumab) show favorable results [52–54]. These results suggest a connection between IL-1*β* and pathological hypertrophy. In line with this notion, the cardiac-specific overexpression of IL-1 can cause myocardial hypertrophy in C57BL/6 N mice [55]. In neonatal rat cardiac myocytes, IL-1*β* induces the reexpression of myocyte hypertrophy-associated fetal genes (*β*-MHC and ANP) [56]. These findings expand our understanding of inflammation and reveal that the cytokine IL-1*β* is a vital inducer of cardiac hypertrophy. As mentioned previously, IL-1*β* maturation is largely dependent on NLRP3 inflammasome activation. The NLRP3 inflammasome is activated and increases caspase-1 and IL-1*β* expression in a rat myocardial hypertrophy model developed by aortic transverse contraction and in human cardiomyocytes treated with angiotensin II [57]. Knockdown of NLRP3 with siRNA or pharmaceutical inhibition of NLRP3 inflammasome activation reverses myocardial hypertrophy markers and myocardial fibrosis-associated protein expression both in vivo and in vitro [57, 58]. In a DCM mouse model induced by streptozotocin (STZ), inhibiting NLRP3 inflammasome activation remarkably improves cardiac function and decreases myocardial hypertrophy induced by hyperglycemia [28]. These findings raise the possibility that targeting NLRP3 inflammasome-mediated myocardial hypertrophy may intervene in DCM.

### 3.2. NLRP3 Inflammasome in Cardiac Fibroblasts

Cardiac fibroblasts (CFs) are the predominant cell type in the cardiac interstitium. They are able to maintain the integrity of the extracellular matrix network, thus preserving geometry and function. Following myocardial injury, quiescent cardiac fibroblasts differentiate into myofibroblasts to express contractile protein *α*-SMA, exhibit proliferation and migration properties, modify extracellular matrix turnover through synthesis and secretion of extracellular matrix proteins, and regulate matrix metabolism [59]. Dynamic phenotypic alterations of CFs direct the reparative response after acute myocardial injury and mediate cardiac fibrosis with chronic diseases. However, dysregulated phenotypic transdifferentiation of CFs is thought to be a crucial mechanism of cardiac remodeling. Beyond these roles, CFs can also serve as sentinel cells to recognize DAMPs and promote NLRP3 inflammasome activation and IL-1*β* generation, thus participating in the inflammatory response of cardiac repair [16, 60].

It was found that the NLRP3 inflammasome in cardiac fibroblasts can lead to adverse myocardial remodeling, increased myocardial stiffness and uncoordinated contraction [61]. Mechanistically, NLRP3 inflammasome activation significantly upregulates the expression levels of *α*-SMA, protein collagen I, and collagen III in the heart tissues of doxorubicin-treated mice, while inhibition of NLRP3 via siRNA suppresses CF proliferation, migration, and collagen secretion [62]. A similar result was obtained in a myocardial infarction rat model; MCC950, a selective NLRP3 inflammasome inhibitor, effectively attenuates the area of whole heart fibrosis and left ventricle collagen volume fraction by downregulating caspase-1 and IL-1*β* expression [63]. The above studies demonstrate that the NLRP3 inflammasome participates in the phenotypic transition of CFs and exacerbates cardiac fibrosis.

Cardiac fibrosis is one of the main structural disorders of DCM. During DCM, the NLRP3 inflammasome aggravates cardiac fibrosis and promotes hyperglycemia-induced CF activation [64]. In a model of type 2 diabetes induced by STZ along with a high-fat diet, the expression of NLRP3, ASC, caspase-1, and IL-1*β* is upregulated in the myocardium, while knockdown of the NLRP3 gene distinctly reduces both the cardiac fibrosis area and synthesis of collagen I and collagen III in the cardiac interstitium [26]. Subsequent studies discovered that rosuvastatin (RSV), a widely used lipid-lowering drug, can improve cardiac function, interstitial fibrosis, and cardiac structural disorders in DCM by inhibiting NLRP3 inflammasome activation, independent of its ability to ameliorate systemic metabolic dysregulation, while these protective effects are attenuated after downregulating NLRP3 [31]. In vitro, primary rat cardiac fibroblasts treated with HG and ATP demonstrate significantly elevated *α*-SMA expression and deposition of collagen I and collagen III via ROS- and P2X7R-mediated rapid stimulation of the NLRP3 inflammasome, followed by increased IL-1*β* and IL-18 levels [65]. Moreover, the underlying molecular mechanisms of the NLRP3 inflammasome in cardiac fibrosis may be related to the interaction between the NLRP3 inflammasome/IL-1*β* axis and the TGF-*β*/Smad signaling pathway [66, 67]. Collectively, these studies indicate that the prolonged expression and activation of the CF NLRP3 inflammasome induced by DM contribute to cardiac fibrosis progression, whereas these pathologies can be eliminated by ablation of cardiac NLRP3 inflammasome activity.

### 3.3. NLRP3 Inflammasome in Endothelial Cells

Coronary artery endothelial cells (CAECs) play an important role in regulating vascular tone and permeability, hemostasis, angiogenesis, and inflammation of the coronary artery [68]. Impaired coronary artery endothelial homeostasis results in the onset and development of DM-related cardiac complications, including coronary artery disease, heart failure, and DCM. At present, vascular endothelial dysfunction has been thought to be one of the key pathological bases and pathogenic mechanisms of DCM [69]. The active NLRP3 inflammasome has been demonstrated to be involved in endothelial dysfunction under diverse pathological stimuli [70]. Endothelial barrier dysfunction, myocardial capillary rarefaction, and a shift in endothelial-mesenchymal transition (EndMT) are three major defects caused by the NLRP3 inflammasome. All three may act either alone or in combination, leading to hyperglycemia-induced endothelial dysfunction and structural remodeling of coronary vessels, thereby promoting the occurrence and development of DCM.

#### 3.3.1. NLRP3 Inflammasome in Endothelial Barrier Dysfunction

The endothelial barrier is a selective permeability barrier of the vascular system formed by endothelial cell monolayers. It controls the exchange of fluids and solutes while limits the passage of xenobiotics or immune cell invasion [71]. The endothelial cell layer and interendothelial junctions are the structural basis of the endothelial barrier [72]. Under pathological conditions, such as inflammation and DM, various mediators act on endothelial cells, leading to endothelial cell death and cell–cell junction disruption. Thus, it leads to endothelial barrier dysfunction and causes vessel hyperpermeability. Vessel hyperpermeability accelerates the leakage of proinflammatory cells and proinflammatory cytokines into the interstitial space and forms a vicious cycle of the inflammatory response, which intensifies cardiomyocyte stiffness and hypertrophy [73]. Recent studies have demonstrated a pivotal role of NLRP3 inflammasome activation in endothelial barrier dysfunction by inducing pyroptosis and disruption of tight junctions [74–77]. In DM, hyperglycemia changes endothelial permeability by inducing endothelial cell pyroptosis in an NLRP3 inflammasome-dependent manner, and inhibition of the NLRP3 inflammasome ameliorates endothelial barrier dysfunction [78, 79]. In addition, increased formation and activation of the NLRP3 inflammasome complex, characterized by increased production of IL-1*β* and caspase-1, downregulate the expression levels of tight junction protein zonula occludens-1/2 (ZO-1/2) in coronary arterial endothelium of STZ-induced diabetic NLRP3+/+ C57BL/6J mice, leading to coronary arterial endothelium barrier integrity wreck and increased endothelial permeability, while NLRP3 deletion eliminates the destruction of the endothelial barrier and restores the expression of tight junction protein [32, 33]. In vitro, inhibition of the NLRP3 inflammasome by puerarin ameliorates endothelial gap junction dysfunction by restoring the expression of the endothelial tight junction protein ZO1/2 in the CAECs of diabetic mice [34]. The underlying mechanism involves the proinflammatory mediators released upon NLRP3 inflammasome activation, including IL-1*β*, IL-18, and high-mobility group protein B1 (HMGB1). IL-1*β* and IL-18 can activate the NF-*κ*B signaling pathway, which promotes the expression of chemokines and adhesion molecules, followed by leukocyte adhesion and endothelial inflammation, ultimately triggering endothelial barrier dysfunction. HMGB1 can directly lead to damage of interendothelial junctions; moreover, it can promote NLRP3 inflammasome activation by binding to receptors (including TLR2, TLR4, and RAGE) to form a feedback loop [32, 80]. Collectively, NLRP3 inflammasome is a key involvement in the regulation of EC functions and the EC inflammatory responses in DM. The development of specific drugs targeting the NLRP3 inflammasome is a promising direction to reduce the morbidity and mortality of DM-related vascular complications (including macrovascular and microvascular disorders).

#### 3.3.2. NLRP3 Inflammasome in Myocardial Capillary Rarefaction

Coronary capillaries actively participate in the important functions of the cardiovascular system and provide a broad endothelial interface for efficient solute and gas exchange. Permanent loss of capillaries leads to cardiac damage [81]. Myocardial capillary rarefaction is a structural defect of the coronary microvasculature that can lead to microcirculation dysfunction. Microcirculation dysfunction, the earliest manifestation of cardiovascular diseases, is consistently observed in DCM patients. Corresponding microvascular dysfunction is evident by the higher vulnerability of the diabetic heart to coronary microcirculation dysfunction [82, 83], and improving coronary microvascular function by fluvastatin treatment or enhancement of angiogenesis could ameliorate cardiac hypertrophy and dysfunction in DCM models [84–86]. These findings suggest that functional and structural alterations in the microvasculature lead to hypoperfusion of the myocardium and thereby exacerbate cardiac energetic deficits during the development of DCM.

Coronary microvascular rarefaction implies an imbalance between vessel destruction and regeneration. Impaired neovascularization by hyperglycemia further contributes to coronary microvascular rarefaction [87, 88]. NLRP3 inflammasome-induced endothelial cell loss and neovascularization impairment are involved in DM-related myocardial capillary rarefaction. NLRP3 inflammasome-related pyroptosis activation inhibits the angiogenic ability of HUVECs by decreasing the expression of CD31, CD34, VEGFA, VEGFR2, ANG2, and TIE2 in the myocardium. These results indicate that NLRP3 inflammasome activation reduces cardiac microvascular density, while NLRP3 inflammasome blockage can alleviate the restrained angiogenic ability [89]. Moreover, in peripheral artery diseases, inhibition of NLRP3 inflammasome activation in endothelial cells promoted angiogenesis and blood perfusion [90–92]. In the above, the NLRP3 inflammasome has been shown to be a key player in the development of angiogenesis in various pathogenic conditions. However, further exploration to validate the molecular mechanism of the NLRP3 inflammasome in angiogenesis is urgently needed. These findings inspired us to ponder the use of antibody-mediated blockade of the NLRP3 inflammasome as a latent therapeutic option against DCM, which merits further exploration.

#### 3.3.3. NLRP3 Inflammasome in Endothelial-Mesenchymal Transition (EndMT)

Endothelial-mesenchymal transition (EndMT) is a complex biological process characterized by the loss of the expression of specific endothelial cell markers in endothelial cells but increased expression of mesenchymal cell markers [93]. During EndMT, endothelial cells lose adherens junction proteins and dissociate from the neatly arranged endothelial layer. Endothelial cell-derived mesenchymal cells migrate to perivascular tissues, thus leading to endothelial dysfunction and aggravating interstitial fibrosis and vascular remodeling. Numerous studies have expanded our knowledge of EndMT and provide evidence for the important role of EndMT under various pathological conditions, especially in cardiac remodeling [93–96]. Diabetes induces the emergence of fibroblasts originating from endothelial cells by EndMT, and the transformed fibroblasts greatly exacerbate cardiac fibrosis and cardiac dysfunction in diabetic cardiomyopathy [97–99]. Suppression of EndMT could prevent diabetic cardiomyopathy in diabetic animal models [100–102]. EndMT has been considered a key link in the interplay between inflammation and cardiac remodeling [103]. Inflammatory conditions are mediated by several mediators of inflammation. Mediators of inflammation mainly include proinflammatory cytokines such as IL-1*β* and TNF-*α*. IL-1*β* has been shown to activate ECs and convert them into activated fibroblasts through an EndMT-based mechanism. Therefore, a close relationship between the EndMT process and inflammation emerges [104]. Activation of the NLRP3 inflammasome leads to cleavage of procaspase-1 into active caspase-1, followed by the processing of pro-IL-1*β*/18 into mature IL-1*β*/18. Furthermore, the most recent study showed that NLRP3 inflammasome activation results in ventilator-induced lung fibrosis by promoting pulmonary endothelial cells to undergo EndMT, while NLRP3 deficiency ameliorates EndMT and pulmonary fibrosis in vitro and in vivo [105], suggesting that the suppression of EndMT by NLRP3 inflammasome deactivation may be a feasible strategy against fibrogenesis. Although the importance of inflammation promoting the EndMT process in cardiac remodeling has attracted extensive attention, most studies are limited to identifying the changes in endothelial and mesenchymal markers in response to proinflammatory cytokines. Therefore, it will be of great significance to clarify the potential molecular regulatory mechanism of inflammatory stimulation induced pathological EndMT in the future.

**Figure 2:**
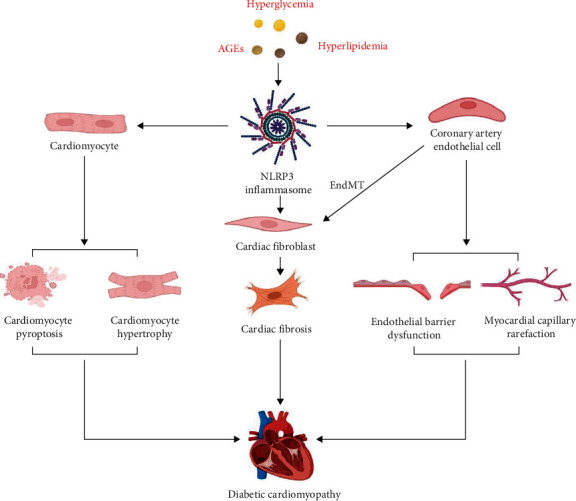
Cell-specific roles of the NLRP3 inflammasome in DCM. The NLRP3 inflammasome in cardiac resident cells, including cardiomyocytes, cardiac fibroblasts, and coronary artery endothelial cells, is activated by DAMPs (e.g., hyperglycemia, hyperlipidemia, and AGEs) in DM. NLRP3 inflammasome activation in cardiomyocytes causes cardiomyocyte cell loss and myocardial hypertrophy and leads to cardiac fibrosis by promoting cardiac fibroblast activation. The NLRP3 inflammasome also contributes to EndMT, endothelial barrier dysfunction, and myocardial capillary rarefaction in coronary artery endothelial cells. All are involved in the pathophysiology of diabetic cardiomyopathy. AGEs: advanced glycation end products, EndMT: endothelial-mesenchymal transition.

The cell-specific roles of the NLRP3 inflammasome in DCM are shown in Figure 2.

## 4. Therapies Targeting the NLRP3 Inflammasome for DCM

Only 9% of the risk of major adverse cardiovascular events (MACE) was eliminated, and the risk of HF was not affected at all in T2D patients after achieving the best possible glycemic control [106], suggesting that optimal glycemic control is not enough to prevent the development of DCM. Based on the aforementioned experimental data, therapeutic interventions against the NLRP3 inflammasome may provide a new strategy for DCM. The complex activation process of the NLRP3 inflammasome provides a variety of targets to inhibit its activation, including suppression of upstream signals inducing NLRP3 inflammasome formation, inhibition of caspase-1 activation and GSDMD cleavage, and blocking of NLRP3 inflammasome-derived inflammatory cytokines [107]. In this section, we review preclinical and clinical studies that target the NLRP3 inflammasome to improve DCM. The therapeutic methods and mechanisms of action on the NLRP3 inflammasome are summarized in Tables 2 and 3.

### 4.1. Pharmacological Treatments

Accumulated studies indicated that pharmacology-based strategy could exhibit the cardioprotective effects under high glucose and high-fat microenvironment. Below are details about these drugs.

#### 4.1.1. Antidiabetic Drugs

Metformin is the most widely used drug for type 2 diabetes mellitus and exerts its antidiabetic activity primarily by reducing gluconeogenesis. In addition to its hypoglycemic effect, it is widely reported that metformin has antitumor, anti-inflammatory, antiaging, and cardioprotective effects. Metformin can alleviate DCM by inhibiting NLRP3 inflammasome activation, which is demonstrated by downregulating the expression of NLRP3, caspase-1, and GSDMD-NT [108]. Moreover, metformin exhibits anti-inflammatory properties, in part through inhibiting NF-*κ*B, which in turn could inhibit the activation of the NLRP3 inflammasome and/or decrease the expression of NLRP3 inflammasome components [139]. However, the effect of metformin has not been evaluated in clinical trials by recruiting DCM patients. Therefore, whether metformin is effective and safe in patients with DCM is still inconclusive.

Glyburide is a widely used oral antidiabetic agent for type 2 diabetes and showed strong effects in reducing left ventricular mass in patients with type 2 diabetes in a network meta-analysis [140]. Glyburide inhibits NLRP3 inflammasome activation in cardiomyocytes [110] and CFs [141] in addition to its antidiabetic activity. Mechanistic explorations have indicated that the underlying mechanism of preventing the assembly of the NLRP3 inflammasome involves glibenclamide-mediated closure of ATP-sensitive potassium channels and suppression of ROS generation [110]. Since glyburide is an established Food and Drug Administration- (FDA-) approved drug with an excellent safety profile, repurposing for DCM may be an appealing strategy.

SGLT-2 inhibitors are a new class of oral hypoglycemic drugs, and their mechanism of action is the inhibition of glucose reabsorption at the level of the proximal tubule. As demonstrated by the results of the EMPA-REG OUTCOMES trial, SGLT-2 inhibitors show great potential cardioprotective effects in improving DCM and anti-inflammatory properties, regardless of hypoglycemic capability [142]. Among them, dapagliflozin attenuates NLRP3 inflammasome activation in BTBR mice and cardiac fibroblasts exposed to LPS. Recent studies have revealed that the mechanism underlying the action of dapagliflozin involves activating the AMPK signaling pathway [112]. Furthermore, SGLT-2 inhibitors significantly reduce the levels of IL-1*β* and IL-18, consistent with reduced activity of the NLRP3 inflammasome, leading to reduced adverse cardiac events in patients with T2D and CVD in a *β*-hydroxybutyrate- (BHB-) dependent manner [143]. Mechanistically, BHB inhibits the NLRP3 inflammasome by preventing K^+^ efflux and reducing ASC oligomerization and speck formation. The inhibitory effects of BHB on NLRP3 are not dependent on chirality- or starvation-regulated mechanisms such as AMPK, reactive oxygen species (ROS), autophagy, or glycolytic inhibition [120]. These results highlight that targeting the NLRP3 inflammasome with SGLT-2 inhibitors may be a promising novel therapeutic strategy for the treatment of DCM.

Linagliptin, a DPP-4 inhibitor, is an oral antihyperglycemic agent in DM treatment that prolongs the half-life of glucagon-like peptide 1 (GLP-1). Linagliptin improved cardiac systolic dysfunction and adverse remodeling by inhibiting NLRP3 inflammasome activation in db/db-infarct mice [114]. DPP-4 inhibitors alleviate NLRP3 inflammasome-mediated inflammatory effects in macrophages through inhibition of the protein kinase C (PKC) pathway, which further results in decreased ROS formation and downregulates the activity of the NLRP3 inflammasome [144]. It has been reported that exendin-4, a GLP-1 analog, reduces ROS formation through the AMPK-TXNIP pathway in type 2 diabetic mice induced by a high-fat diet to attenuate NLRP3 inflammasome activity, thereby improving cardiac dysfunction and remodeling [145]. Linagliptin may represent an attractive strategy for the treatment of DCM. More clinical trials are required to determine the functional role and safety profile of linagliptin in DCM.

#### 4.1.2. Natural Compounds

Research on the cardioprotective effects of natural compounds against the NLRP3 inflammasome is booming in preclinical and clinical studies [146]. To date, 6 active agents, namely, ginsenoside, puerarin, betulin, gypenosides, tilianin, and syringin, have been confirmed to ameliorate DCM through inhibition of the NLRP3 inflammasome [147]. Diverse natural compounds exhibit notable inhibitory effects on anticardiac inflammatory responses by modulating the priming phase of the NLRP3 inflammasome in diabetic myocardial tissue, mainly through suppressing NF-*κ*B- or ROS-mediated signaling. Certain natural compounds exert antioxidant effects and concomitantly inhibit the NLRP3 inflammasome by promoting the transcriptional activity of nuclear factor-erythroid 2-related factor 2 (Nrf2) in vitro and in vivo. Medicinal plant extracts suppress NF-*κ*B transcriptional activity and decrease the levels of NLRP3 inflammasome components, subsequently improving cardiac structure and function in diabetic rodents. Natural compounds or herbal medicines shed light upon the future direction of pharmacological research and provide a novel target for the treatment of DCM. However, the long-term effects, safety files, and clinical relevance of natural compounds are warranted to discover with regard to DCM.

#### 4.1.3. IL-1*β* Antagonists

As a member of the IL-1 family of cytokines, the evidence supporting the role of IL-1*β* in adversely affecting myocardial contractility and cardiac remodeling under DCM is straightforward [148]. The CANTOS trial highlighted the importance of direct anti-inflammatory therapies targeting the NLRP3 inflammasome-derived inflammatory cytokine IL-1*β* in secondary cardiovascular disease prevention [149]. Canakinumab, an IL-1*β* neutralizing antibody, alleviated the burden of DCM [19]. Gevokizumab appears to be most similar to canakinumab due to IL-1*β* selectivity and a pharmacokinetic profile and is not currently approved for any indications in the US [148]. In the REDHART and D-HART trials, anakinra, a recombinant IL-1 receptor antagonist, promoted a significant improvement in cardiorespiratory fitness and improved the aerobic exercise capacity of patients with HF [52, 53]. In addition, anakinra can improve endothelial dysfunction in diabetic rat models [150]. Rilonacept, a decoy receptor that binds IL-1*β* and IL-1*α*, also displayed potential therapeutic effects in cardiovascular diseases [151], but there was only one clinical trial on cardiovascular diseases, and the subjects were patients with recurrent pericarditis [126]. These antagonists have shown great cardioprotective potential in preclinical and clinical studies but have not yet been investigated in the context of DCM. Hence, IL-1*β* blockers in DCM are still in their infancy and may represent a unique opportunity to quench the inflammatory response following DM by selectively inhibiting a single apical mediator in the cascade, especially for patients with DCM combined with chronic inflammatory diseases.

#### 4.1.4. Other Potential NLRP3 Inflammasome Inhibitors

Studies on the role of the NLRP3 inflammasome in DCM patients are scarce, let alone clinical trials of NLRP3 inflammasome inhibitors, indeed, with the exception of OLT117, which is currently being investigated in a phase I clinical trial for the treatment of heart failure. Besides, colchicine, a nonspecific NLRP3 inflammasome inhibitor, has been recently shown great cardioprotective potential, but whether the effects are mediated by the NLRP3 inflammasome and/or IL-1 signaling remains to be determined [152]. An increasing number of reports reveal that MCC950, parthenolide, BAY 11-7082, and INF39 demonstrated beneficial effects on inflammatory diseases by derangement of the combination between NLRP3 and ASC or NLRP3 ATPase inhibition [153–155]. Moreover, the caspase-1 inhibitor pralnacasan exerted beneficial therapeutic effects on inflammatory diseases [156, 157]. These treatments have shown a strong anti-inflammatory effect via inhibition of the NLRP3 inflammasome. This beneficial effect is based on inflammatory disease models, but these inhibitors might represent interesting directions for further studies in the treatment of DCM and can be further translated into clinical practice.

### 4.2. Nonpharmacological Inhibitions

Recent findings suggest nonpharmacological treatments such as nutritional interventions, microbiota-targeted therapies, and exercises.

#### 4.2.1. Nutritional Interventions

It has long been noted that the Western dietary pattern is one of the main risk factors for DM and diabetic complications. Nutritional interventions are a cornerstone recommended for diabetic patients and have been established as an effective approach for the management of DM and DCM by the American Diabetes Association (ADA) and cardiovascular experts. Complex nutraceutical programs have been confirmed to have preventive and therapeutic utility in a wide range of diseases in which NLRP3 inflammasome activity plays a mediating role, such as COVID-19, chronic kidney disease, and metabolic disorders [158, 159]. A ketogenic diet is a high-fat, low-carbohydrate, and adequate-protein formulation [160], which has an antidiabetic effect and cardioprotective effect and can decrease NLRP3 inflammasome–induced IL-1*β* and IL-18 release via BHB [120, 121]. The ketogenic diet has gradually become a selective dietary intervention option for cardiometabolic diseases. When the hearts of db/db mice have been challenged with hyperglycemia, the ketogenic diet has been shown to have a protective effect on the heart, partly due to its anti-inflammatory activity [121]. However, there are also studies with conflicting or controversial findings and opinions [161, 162]. These results suggested that a ketogenic diet might lead to maladaptive cardiac metabolic modulation and lipotoxicity and deteriorate diabetic cardiomyopathy [163]. Given this, the possible role of ketogenic diet in DCM remains controversial and warrants more studies for elucidation.

Recent studies have shown that a Mediterranean diet can exert anti-inflammatory properties and improve cardiac function in DCM. A Mediterranean diet consists of fish, olive oil, fruits, vegetables, whole grains, legumes or nuts, and moderate consumption of alcohol, most commonly red wine [164]. As shown in the CORDIOPREV trial, a Mediterranean diet leads to markedly reducing the burden or even preventing the development of DM and cardiometabolic risk, which may depend on genetic variation in the NLRP3 inflammasome [122]. In the MEDIT-AHF clinical trial, a Mediterranean diet was also found to decrease the rehospitalization rates of acute cardiac insufficiency [123]. A Mediterranean diet, especially rich in phenolic compounds, has a strong antioxidant capacity to inhibit NLRP3 inflammasome-mediated inflammation and cell loss [165]. Nutritional interventions are currently commercially available and can be assumed to be fully absorbed and physiologically active in the prescribed oral dosage regimen and have a reasonable and clear mechanism of action. Therefore, it may have huge potential clinical utility in controlling the activation of the NLRP3 inflammasome.

#### 4.2.2. Microbiota-Targeted Therapies

The gut microbiome performs multiple functions in the host, including the synthesis of bioactive products, metabolism of dietary compounds, and immune regulation [166]. Changes to the microbiota (including imbalances in quantity and/or quality among the phyla and generation of certain bacterial metabolites) have been recognized as important contributors to the pathophysiology of DM and cardiovascular diseases [167]. Gut microbiota dysbiosis provokes changes in intestinal wall permeability and enhances the secretion of bacterial metabolites, the diffusion of metabolites such as TMAO or bacterial endotoxins such as LPS into the host bloodstream interacting with receptors on the surface of a multitude of cell types, including immune cells, cardiomyocytes, and cardiac fibroblasts, thereby favoring the emergence of inflammation in the host, activating the NLRP3 inflammasome, and causing deleterious effects on the myocardium [168, 169]. Beneficial modulation of the gut microbiota has been demonstrated as a noninvasive treatment for NLRP3 inflammasome-related diseases. Probiotics ameliorated chronic metabolic inflammation and inhibited NLRP3 inflammasome activation by modulating the gut microbiota in high-fat diet-fed animal models [170, 171]. Moreover, probiotics show cardioprotective effects in various cardiovascular diseases [172, 173]. As demonstrated in a randomized, double-blind, placebo-controlled pilot trial, probiotic administration (Saccharomyces boulardii) has been shown to improve cardiac function and reduce inflammatory biomarkers compared with the placebo group [174]. Probiotics play a cardioprotective role by reducing DM-related inflammation, hypertrophy, and fibrosis in DCM experimental animals. The potential mechanism is linked to the inhibition of TLR4 signaling pathways, a priming signal for the NLRP3 inflammasome [125]. Targeted inhibition of the NLRP3 inflammasome through probiotics may have the potential to prevent or suppress diabetic cardiomyopathy. Therefore, more experimental and clinical trials are needed to map its safety and efficacy in the treatment of DCM. Fecal microbiota transplantation (FMT), a procedure in which feces from a healthy donor is implanted into the gastrointestinal tract of another patient, helps restore the balance of healthy bacteria and regulate the immune and inflammatory responses in recipients. Such therapy is a Food and Drug Administration-approved technique for the treatment of Clostridium difficile infection [175]. Recently, attempts have been made to apply FMT to the treatment of diseases such as metabolic syndrome and inflammatory diseases [176]. In addition, microbial dysbiosis causes impaired glucose tolerance and enhanced NLRP3 inflammasome activity, leading to an increase in atrial fibrillation (AF) susceptibility in elderly patients and animal models. FMT successfully inhibited the cardiac NLRP3 inflammasome and ultimately attenuated increased AF susceptibility and cardiac fibrosis, suggesting that FMT-targeted inhibition of the NLRP3 inflammasome is a new innovative therapeutic option for metabolic diseases and cardiovascular diseases [177, 178]. Moreover, it is worth noting that microbiota-targeted therapies seem to be mainly driven by abundance-based microbiota composition analysis, in which microbiota members highly related to beneficial phenotypes are the focus of interest. However, the keystone commensal may be low abundance microbial community members and is usually not easy to detect by current conventional sequencing in-depth analysis.

#### 4.2.3. Exercise Intervention

Regular physical activity could effectively lower the HbA1c level and gradually improve cardiac function; so, it is recommended as the basic treatment for diabetes patients, especially for patients with DCM. Moreover, exercise intervention benefits health partly by suppressing inflammation. Aerobic training attenuated the increased NLRP3 inflammasome activity, as demonstrated by reduced systemic and local IL-1*β* and IL-18 levels [179]. Recent studies indicated that aerobic exercise reversed cardiac dysfunction by mitigating the NLRP3 inflammasome to abrogate myocardial inflammation and pyroptosis, and the underlying mechanism may be partly through the P2X7R-inflammasome axis [180]. Regular aerobic exercise intervention is an effective and economical method of prevention and treatment for alleviating DCM by regulating the NLRP3 inflammasome. However, the inhibitory effect of exercise intervention on the NLRP3 inflammasome depends on the exercise duration and intensity [181]. Therefore, the exercise regimen needs to be individualized, as there are no guidelines in this regard, further research is needed.

## 5. Conclusions and Future Directions

The pathogenesis of DCM is complex and involves many distinct pathways. Clinical and preclinical studies demonstrate that myocardial inflammation is an important pathogenic factor in diabetes-induced cardiac dysfunction, and alleviation of myocardial inflammation is closely associated with preserved cardiac function. The activation of the NLRP3 inflammasome in local cardiac cells serves as a trigger for inflammation in DCM. It is activated under diabetes conditions that promotes caspase-1 autocleavage, and the maturation of pro-IL-1*β*/18, IL-1*β*, and IL-18 is secreted into the extracellular space to participate in the subsequent inflammatory response. Meanwhile, it also leads to the occurrence of GSDMD-mediated programmed cardiac resident cell death, called pyroptosis. Therefore, the NLRP3 inflammasome plays a critical role in the development and progression of DCM, which provides new insights into the molecular mechanisms of DCM as well as potential therapeutic targets for its prevention and treatment. At present, potential therapeutic strategies targeting the NLRP3 inflammasome may be efficacious in the prevention of DCM and may broaden the therapeutic field in DCM, which are illustrated in this review.

Of note, the NLRP3 inflammasome, a critical player in the immune response, is essential for the recognition and elimination of danger. However, hyperactivation of the NLRP3 inflammasome contributes to unresolved inflammation, consequently leading to tissue damage. Undeniably, it has been clearly indicated that the dysregulation of the NLRP3 inflammasome plays a key role in the pathogenesis of diabetes, obesity, cardiovascular diseases, cancer, etc. Nevertheless, increasing evidence suggests that its activation could exert a beneficial effect in some forms of cancer, inflammatory diseases, and glucose metabolism and increase plaque stability in atherosclerosis, which reminds us that the beneficial role of the NLRP3 inflammasome should not be ignored [182–186]. Therefore, therapeutic strategies to inhibit the NLRP3 inflammasome require further investigation, especially in diseases with complex etiologies that respond poorly to treatment. It is critical to improve our mechanistic understanding of the divergent roles of the NLRP3 inflammasome in disease-specific contexts. Thus, detailed research via the application of cell- or tissue-specific NLRP3 knockout is essential to completely understand the specific functions and immune-inflammatory pathways of the NLRP3 inflammasome under disease-specific or cell-specific conditions, as well as to identify novel specific inhibitors, helping to balance the beneficial and detrimental functions of this key player in clinical settings.

## Figures and Tables

**Table 1:** The NLRP3 inflammasome in DCM.

Conditions	Models	NLRP3 inflammasome	Biological outcomes	Diabetic cardiomyopathy	Year of publication	References
High-fat diet with STZ	Sprague-Dawley rats	Activation	Cardiomyocyte pyroptosis	Promoting	2020	[24]
High glucose (35 mM glucose)	Human cardiomyocytes	Activation	Cardiomyocyte pyroptosis	Promoting	2016	[25]
High-fat diet with STZ, knockdown NLRP3	Sprague-Dawley rats	Inhibition	Cardiomyocyte pyroptosis	Improving	2014	[26]
High glucose (33.3 mM glucose), NLRP3 gene silencing	H9c2 cardiomyocytes	Inhibition	Cardiomyocyte pyroptosis	Improving	2014	[26]
High-fat diet with STZ	Wistar rats	Activation	Cardiomyocyte pyroptosis	Promoting	2014	[27]
High glucose (30 mM and 50 mM glucose), miroRNA-30d mimic	Neonatal cardiomyocytes	Inhibition	Cardiomyocyte pyroptosis	Improving	2014	[27]
High-sucrose/high-fat diet with STZ	C57BL/6 mice	Activation	Cardiomyocyte pyroptosis and hypertrophy	Promoting	2020	[28]
High glucose (30 mM glucose)	HL-1 cells	Activation	Cardiomyocyte pyroptosis	Promoting	2020	[28]
High-fat diet with STZ	C57BL/6 J mice	Activation	Cardiomyocyte pyroptosis and hypertrophy	Promoting	2022	[29]
Palmitic acid (200 *μ*M)	H9c2 cardiomyocytes	Activation	Cardiomyocyte pyroptosis and hypertrophy	Promoting	2022	[29]
High glucose (30 mM glucose), si-Kcnq1ot1	Primary cardiac fibroblasts	Inhibition	Cardiac fibroblast pyroptosis, cardiac fibrosis	Improving	2018	[30]
High-fat diet with STZ, knockdown NLRP3	Sprague-Dawley rats	Inhibition	Cardiac fibrosis	Improving	2014	[31]
High-fat diet with STZ	C57BL/6 J NLRP3^−/−^ mice	Inhibition	Abolishing endothelial dysfunction	Improving	2016	[32]
High glucose (30 mM glucose), NLRP3 gene silencing	Mouse vascular endothelial cell	Inhibition	Preventing tight junction disruption	Improving	2016	[32]
High-fat diet	C57BL/6 J NLRP3^−/−^ mice	Inhibition	Abolishing endothelial dysfunction	Improving	2015	[33]
High glucose (30 mM glucose) with puerarin	Mouse vascular endothelial cell	Inhibition	Preventing tight junction disruption	Improving	2019	[34]

**Table 2:** Therapies targeting NLRP3 inflammasome investigated in the context of DCM.

Therapies		Pathways or mechanisms	Study types	Models	Animals and cells	Conditions	Doses and duration	Refs.
Metformin		ROS inhibition, NF-*κ*B activity limitation	In vitro, in vivo	Diabetic cardiomyopathy	C57BL/6 mice, primary cardiomyocytes Wistar rats	STZ, high glucose (25 mM glucose) STZ	200 mg/kg/day for 8 w, 2 mM for 24 h 200 mg/kg/day for 6 w	[108, 109]
Glyburide		Closure of potassium channel, ROS inhibition	In vivo	Atrial remodeling induced by DM	Japanese long-ear white rabbits	Alloxan	2 mg/kg/day for 6 w	[110, 111]
SGLT-2 inhibitors		ROS inhibition	In vitro, in vivo	Diabetic cardiomyopathy	BTBR ob/ob mice, cardiofibroblasts generated from BTBR ob/ob mice	—	1 mg/kg/day for 8 w or 1.5 mg/kg/day for 12 w, 0.4 *μ*M for 16 h	[112, 113]
DPP-4 inhibitors		ROS inhibition	In vitro, in vivo	Diabetic cardiomyopathy Ischemia–reperfusion and myocardial infarction model	BTBR ob/ob mice db/db mice, human cardiomyocytes and cardiofibroblasts	- High glucose (25 mM glucose)	10 mg/kg/day for 8 w	[112, 114]
Natural compounds	Puerarin	ROS inhibition, NF-*κ*B activity limitation	In vitro	Endothelial barrier dysfunction induced by DM	Mouse vascular endothelial cell (mMVECs)	High glucose (30 mM glucose)	50 *μ*M for 24 h	[34]
	Syringin and tilianin		In vitro, in vivo	Diabetic cardiomyopathy	Sprague-Dawley rats, H9c2 cardiomyocytes	High-fat diet with STZ, high glucose (33 mM glucose)	Syringin 50 mg/kg/day and tilianin 60 mg/kg/day for 8w, syringin 15 *μ*M, and tilianin 10 *μ*M for 48 h	[45]
	Ginsenoside Rg1		In vivo	Diabetic cardiomyopathy	Wistar rats, C57BL/6 J mice	STZ	20 mg/kg/day for 8 w	[115, 116]
	Gypenosides		In vitro, in vivo	Diabetic cardiomyopathy	Sprague-Dawley rats, H9c2 cardiomyocytes	High-fat diet with STZ, high glucose (35 mM glucose)	200 mg/kg/day for 8 w, 400 mg/L for 48 h	[117]
	Betulin		In vitro, in vivo	Diabetic cardiomyopathy	C57BL/KsJ db/db mice, H9c2 cardiomyocytes	High glucose (30 mM glucose)	40 mg/kg/day for 12 w, 40 *μ*M for 24 h	[118]
MCC950		NLRP3-ASC oligomerization blocking	In vitro, in vivo	Cardiac fibrosis induced by DM	Sprague-Dawley rats, primary neonatal rat cardiac fibroblasts	STZ, high glucose (25 mM glucose)	3 mg/kg/day for 8 w	[119]
Ketogenic diet		BHB	In vitro, in vivo	Diabetic cardiomyopathy	C57BL/KsJ db/db mice, neonatal myocytes generated from Sprague-Dawley rats	High glucose(30 mM glucose)	Ketogenic diet for 8 w, BHB 10 mM for 1 h	[120, 121]
Mediterranean diet		ROS inhibition	Clinical	Diabetic patients	Diabetic patients	—	—	[122, 123]
Exercise intervention		P2X7R	In vitro, in vivo	Myocardial inflammation and myocardial remodeling induced by high-fat diet	Sprague-Dawley rats, H9c2 cardiomyocytes	High-fat diet, palmitic acid (200 *μ*M)	Exercise intervention for 12 w	[124]
Microbiota-targeted therapies		NF-*κ*B activity limitation	In vivo	Diabetic cardiomyopathy	Wistar rats	STZ	1 × 10^9^ CFU/rat/day for 4w	[125]

**Table 3:** NLRP3 inflammasome inhibitors currently not investigated in the context of DCM.

Inhibitors	Targeted pathways or pathological mechanisms	Study types	Diseases/models	References
Anakinra	IL-1*β* blockade	Clinical	Heart failure	[52, 53]
Canakinumab	IL-1*β* blockade	Clinical	Diabetes mellitus and high cardiovascular risk	[54]
Rilonacept	IL-1*β* blockade	Clinical	Pericarditis	[126]
CY-09	NACHT ATPase inhibitor: binds walker A motif	In vivo	Myocardial infarction	[127]
Parthenolide	NF-*κ*B activity limitation, caspase-1 inhibitor	In vitro, in vivo	Cardiac transplant ischemia and reperfusion injury	[128]
INF39	NACHT ATPase inhibitor	In vitro, in vivo	T2DM	[129]
BAY11-7082	NLRP3 NACHT domain binding	In vitro, in vivo	Myocardial infarction	[130]
OLT1177	NACHT ATPase inhibitor	In vivo, clinical	Ischemia reperfusion injury, heart failure	[131–133]
Tranilast	NLRP3 NACHT domain binding	In vitro, in vivo	Myocardial infarction, atherosclerosis	[134, 135]
Pralnacasan	Caspase-1 inhibitor	In vitro, in vivo	Myocardial infarction	[136]
YVAD	Caspase-1 inhibitor	In vitro, in vivo	High glucose and hypoxia/reoxygenation injury, cardiac inflammation	[137, 138]
